# Effects of Quinia on Eye and Ear

**Published:** 1883-04

**Authors:** 


### EFFECTS OF QUINIA ON EYE AND EAR.

Clinical investigations have placed the fact beyond doubt that it is not entirely uncommon to see inflammation of the retina or its blood-vessels, and also of the middle and internal ear, following the administration of large doses of this drug. The cases to illustrate this point are too numerous to be explained away, and I believe that argument has ceased against the credibility of the belief that the primary effect of moderately large doses of Sulphate of Quinine is a congestion of some parts of the ear and of the retina. There remain a few who deny that the effects of Quinine are anything but evanescent, but the cases published—first by myself,* and subsequently by Voorhies, De Wecker, Gruening, Buller, Knapp, and others—have finally settled the point that in rare instances deafness and blindness may be caused by large doses of Quinine. Dr. Baldwin, of Alabama, has also published a pamphlet (not at this moment accessible to me), in which he has shown the dire results that have sometimes occurred from the injudicious administration of Quinine, especially in our Southern States.—*Dr. Roosa in Medical Record.*

---

* Archives of Ophthalmology, vol. viii, p. 392; vol. ix, p. 41.

